# Functional and evolutionary analyses of the miR156 and miR529 families in land plants

**DOI:** 10.1186/s12870-016-0716-5

**Published:** 2016-02-03

**Authors:** Edna Gicela Ortiz Morea, Eder Marques da Silva, Geraldo Felipe Ferreira e Silva, Guilherme Targino Valente, Carlos Hernan Barrera Rojas, Michel Vincentz, Fabio Tebaldi Siveira Nogueira

**Affiliations:** Laboratório de Genetica Molecular do Desenvolvimento Vegetal, Departamento de Ciencias Biológicas, Escola Superior de Agricultura ‘Luiz de Queiroz’, Universidade de Sao Paulo, Avenida Padua Dias, 11, 13418-900 Piracicaba, Sao Paulo Brazil; Departamento de Genética, Instituto de Biociencias, Universidade Estadual Paulista (UNESP), Botucatu, Sao Paulo Brazil; Departmento de Bioprocessos e Biotecnologia, Universidade Estadual Paulista (UNESP), Botucatu, Sao Paulo Brazil; Centro de Biologia Molecular e Engenharia Genetica (CBMEG), Universidade Estadual de Campinas, Campinas, Sao Paulo Brazil

**Keywords:** miR156, miR529, Evolution, Arabidopsis, Eudicots

## Abstract

**Background:**

MicroRNAs (miRNAs) are important regulatory elements of gene expression. Similarly to coding genes, miRNA genes follow a birth and death pattern of evolution likely reflecting functional relevance and divergence. For instance, miRNA529 is evolutionarily related to miRNA156 (a highly conserved miRNA in land plants), but it is lost in *Arabidopsis thaliana*. Interestingly, both miRNAs target sequences overlap in some members of the *SQUAMOSA promoter-binding protein like* (*SPL*) family, raising important questions regarding the diversification of the miR156/miR529-associated regulatory network in land plants.

**Results:**

In this study, through phylogenic reconstruction of miR156/529 target sequences from several taxonomic groups, we have found that specific eudicot SPLs, despite miRNA529 loss, retained the corresponding target site. Detailed molecular evolutionary analyses of miR156/miR529-target sequence showed that loss of miR529 in core eudicots, such as Arabidopsis, is correlated with a more relaxed selection of the miRNA529 specific target element, while miRNA156-specific target sequence is under stronger selection, indicating that these two target sites might be under distinct evolutionary constraints. Importantly, over-expression in Arabidopsis of *MIR529* precursor from a monocot, but not from a basal eudicot, demonstrates specific miR529 regulation of *AtSPL9* and *AtSPL15* genes, which contain conserved responsive elements for both miR156 and miR529.

**Conclusions:**

Our results suggest loss of functionality of *MIR529* genes in the evolutionary history of eudicots and show that the miR529-responsive element present in some eudicot *SPLs* is still functional. Our data support the notion that particular miRNA156 family members might have compensated for the loss of miR529 regulation in eudicot species, which concomitantly may have favored diversification of eudicot *SPLs*.

**Electronic supplementary material:**

The online version of this article (doi:10.1186/s12870-016-0716-5) contains supplementary material, which is available to authorized users.

## Background

MicroRNAs (miRNAs) are small RNAs important to transcriptional and post-transcriptional regulation in animals, plants, and viruses. MiRNAs bind complementarily to their target mRNA sequences, leading to translational repression, RNA degradation, or RNA cleavage [1]. Most plant miRNAs are encoded by gene families, and mature miRNAs often have multiple target genes with similar complementary motifs in their mRNAs. The almost perfect complementarity between miRNAs and targets facilitates computational prediction and could be due to their evolutionary origins. One well-accepted model for *MIR* gene evolution is the inverted duplication of target gene sequences in plant genomes. These duplicated regions become *MIR* genes over time through sequence variations, which permit the generation of hairpin-like transcripts to become substrates for DICER-LIKE enzymes [2]. Another interesting model suggests that *MIR* genes can randomly originate from various inverted repeats throughout the genome, independently of target gene duplications [3–6]. For instance, recent evidences indicate that *MIR* genes may be generated as a result of transposon activity [7, 8].

It has been recently shown that evolutionary patterns of miRNA genes (including lineage-specific gain or loss) can potentially influence the evolution of their target genes [9]. Moreover, synonymous codons in target genes have been found near conserved miRNA target sites in at least four plant genomes, indicating selection constraint on synonymous codons for efficient miRNA binding and proper miRNA function [10]. Several miRNAs are conserved throughout large evolutionary distances from embryophyta to core rosids. However, some miRNAs appear to be species or lineage specific [11–13]. For instance, miR156 is conserved in all Angiosperms studied thus far. Interestingly, miR156 is correlated at the nucleotide level with miR529, sharing 14–16 nt [12]. Curiously, although both miRNAs have a common ancestor in embryophytes, miR529 seems to have been lost in some taxonomic groups, including core eudicots such as *Arabidopsis thaliana* [12–14]. Both miRNAs share target genes, which are members of the *SQUAMOSA promoter-binding protein like* (*SPL*) family [15]. *SPL* genes are plant-specific transcription factors defined by a highly conserved region of 76 amino acids called SBP domain [16], and by their crucial and widespread functions in development [17–23]. *SPL* genes can be roughly separated into two major groups–long and short–the latter containing responsive elements for miR156 and, in some species, for miR156 and miR529. Interestingly, sites for miR156 reside in coding as well as untranslated regions of target sequences, whereas miR529 binding sites are chiefly located in coding regions and overlap with miR156 sites [24, 25].

In plant lineages containing both miRNA genes, differential expression of miR156 and miR529 in vegetative and reproductive organs/tissues might have favored lineage-specific retention of miR156/529 sequence variants in their genomes [26]. Another possibility is that the combinatory action of miR156 and miR529 leads to the regulation of distinct targets in specific lineages, such as in *Physcomitrella patens* [27]. Recently, the evolutionary differences between these two miRNA families in monocot species have been investigated [25], but the consequences of miR529 loss in core eudicots such as Arabidopsis are not yet clear. A broader evolutionary analysis of *MIR156* and *MIR529* genes and their targets including eudicot species should offer valuable insight into this issue.

In this study, we extended our knowledge regarding evolutionary and functional divergences between miR529 and miR156 regulation on their conserved targets. Through phylogenic reconstruction of target sequences, we confirmed that specific eudicot *SPL* family members retained miR529-target sites, independently of the presence of *MIR529* genes in their genomes. Loss of miR529 function in eudicots is correlated with a more relaxed selection of the miRNA529-specific target element, while miRNA156-specific target sequence is under stronger selection. Additionally, we showed that *A. thaliana* plants overexpressing *MIR529* precursor from a monocot, but not from a basal eudicot, display similar phenotypes as the *spl9;spl15* mutant due to the specific down-regulation of these miR156/529-targeted *SPLs*. Based upon functional and evolutionary analyses, we proposed that the loss of *MIR529* genes might have favored diversification of *SPL*s in eudicot species. It is also possible that new miR156 family member(s) have replaced miR529 functions in eudicots.

## Results and discussion

### Sequence and phylogenetic analyses reveal that a miR529-responsive element in *SPL* genes is broadly conserved across land plants

**Fig. 1:**
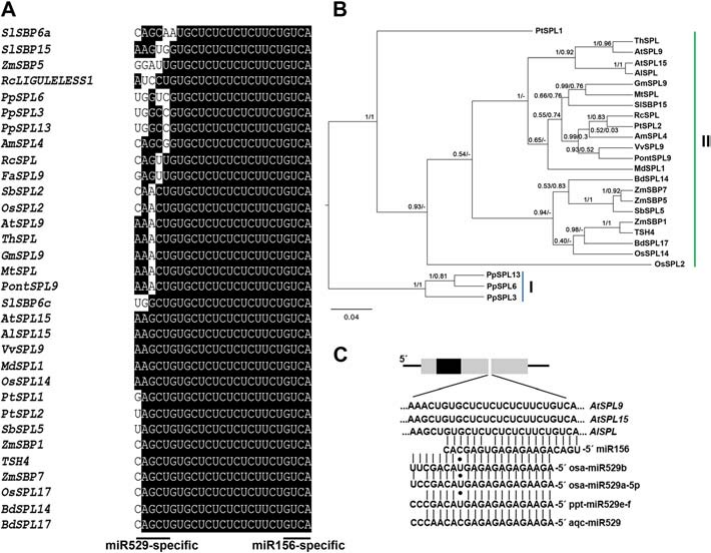
Alignment and phylogeny of SPLs containing the miR156/miR529-response element. **a** Alignments of the 25-nt miR156/529-response element were done using ClustalW (http://www.phytozome.net/). Black color denotes conserved nucleotides. **b** Rooted ML phylogenetic tree depicts the relationship between SPLs containing the miR156/529-response element from representative species. The numbers indicate branch support. Left numbers, posterior probability branch support. Right numbers, aLRT branch support (see Methods). “-”, indicates that a particular branch is not observed in Bayesian or maximum-likelihood trees. Blue line comprises moss SPLs and green line encompasses monocot and eudicot SPLs. **c** Target sites for miR156 and miR529 in *A. thaliana SPL9* and *SPL15* and *A. lyrata SPL* transcripts. At, *Arabidopsis thaliana*; Al, *Arabidopsis lyrata*; Th, *Thellungiella halophila*; Vv, *Vitis vinifera*; Sl, *Solanum lycopersium;* Md, *Malus domestica*; Fa, *Fragaria ananassa*; Pt, *Populus trichocarpa*; Gm, *Glycine max*; Mt, *Medicago truncatula*; Pont, *Poncirus trifoliata*; Am, *Antirrhinum majus*; Rc, *Ricinus communis*; Zm, *Zea mays*; Os, *Oryza sativa*; Bd, *Brachypodium distachyon*; Aqc, *Aquilegia coerulea*; Pp, *Physcomitrella patens*. Accession numbers are given in Additional file 1

*MIR529* genes are present in genomes of a number of species across land plants. Accordingly, in such species, transcripts of a subset of *SPL* family have responsive elements for both miR156 and miR529 [24]. To get a better view of the extent to which a miR529-responsive element is conserved in land plants, we searched for miR529-responsive elements in *SPL* genes from species with publicly available genome sequences, including those species in which miRNA529 is absent in their genomes or in transcribed sequences identified thus far. High-confidence prediction of miRNA targets was performed by psRNATarget based on sequence complementarity and evolutionary conservation [28]. We collected conserved mature miR529 sequences from three species (*Physcomitrella patens*, *Oryza sativa*, and *Aquilegia coerulea*), which are available in miRbase v. 21 (http://www.mirbase.org/) and shown in Fig. 1. We then employed more stringent cut-off threshold for the Maximum expectation (E) parameter (range 0–2.0; [28]) to minimize false positive target prediction (data not shown). Surprisingly, we found *SPL* genes from several species that retain a highly conserved and overlapping responsive element (25 nt in length) for both miRNAs (namely miR156/529-responsive element; Fig. 1a and Additional file 1), independently of the presence of *MIR529* genes in their genomes. This suggests that, whereas *MIR529* and *MIR156* genes have undergone distinct evolutionary fates [25], their mutual targets (which contain the miR156/529-responsive element) have been more conservative even in species which apparently have lost *MIR529* genes. For instance, the miR156/529-responsive element in eudicot *SPLs* resides only in coding regions, similarly to what is observed in monocots and bryophytes (Additional file 1; [24]).

To further explore the evolutionary relationship of miR156/529 common targets, phylogenetic inference of *SBP-box* genes containing the miR156/529-responsive element was estimated using maximum-likelihood (ML) and Bayesian inference methods. The percentage of pairwise identity of well-aligned sequence blocks (see Methods) was 73.3 %, and the substitution saturation test reported that the alignment was not saturated (data not shown). We observed two groups of SPLs in our consensus tree (Fig. 1b). Group I included known miR156/529 *SPL* targets in bryophyte, whereas group II contained various monocot and core eudicot *SPLs* harboring conserved binding sites for miR156/529. This analysis indicated that *SPLs* containing miR156/529 target sites have a common origin in land plants (Fig. 1b). *A. thaliana SPL9* and *SPL15* are closely related and most likely form a pair of paralogous genes [29, 30]. Accordingly, both *SPL9* and *SPL15* as well as their orthologs retained the miR156/529-responsive element (Fig. 1b and c).

It has been proposed for monocot species and *P. patens* that *SPLs* containing miR156/529 sites evolved conservatively with a slow rate when compared with *SPLs* harboring only the miR156-responsive element [24]. To further elucidate the evolutionary fates of eudicot *SPLs* containing the miR156/529-responsive element, we analyzed two blocks in SPL sequences: “SBP domain” block, which contains nucleotides of the SBP domain [16] plus few nucleotides upstream/downstream, and “target site” block, which contains nucleotides that comprise both miR156/529-responsive elements (see Methods). For the “SBP domain” block, we estimated nonsynonymous (Ka) and synonymous substitution (Ks) ratios (Ka/Ks) of representative *SPLs*. We chose Arabidopsis as a representative eudicot due to the fact that, even after extensive sequencing efforts, precursors or canonical mature sequences of miR529 have not been found in either *A. thaliana* or its closest relative *A. lyrata* [31]. Pairwise alignments of best-aligned blocks (249 to 2061 nt) among *SPL* genes from *A. thaliana*, monocots, and *P. patens* indicated that the “SBP domain” block is under purifying selection for all comparisons, reflecting functional constraints, which is expected since SBP is likely a DNA binding domain (Additional file 2; [16]).

**Fig. 2:**
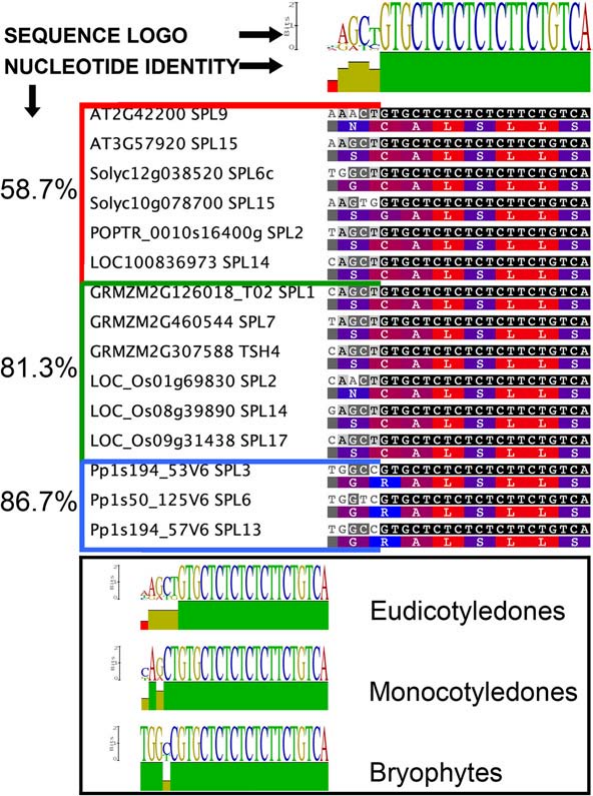
DNA Alignments of miR156 and miRNA156/529 Target Sites. Amino acids coded by miR156/529 target site are shown. Amino acids are colored according to their hydrophobicity; Red box, eudicot sequences; Green box, monocot sequences; Blue box, bryophyte sequences. Percentage of nucleotide identity for the 5′ four nucleotides is shown at the side of each colored box. Black lines underneath the topmost logo indicate miRNA binding sites. Box at the bottom shows logos constructed based on alignments of each plant group. Accession numbers are given in Additional file 1

Given that “target site” blocks are short (25 nt) and could give rise to unrealistic Ka/Ks ratios, we decided to compare their alignment more directly (Fig. 2). Considering the five first bases restricted to the miR529 target site, nonsynonymous substitutions including amino acids with different physicochemical features were found. In contrast, such drastic changes did not occur for the nucleotides specifying miR156 target site (Fig. 2). These observations indicated that different regions of the “target site” block might be under distinct evolutionary constraints. In fact, the bases that diverged more in the “target site” block specifically correspond to the miR529 target site (Fig. 2). Therefore, it is possible that loss of miR529 regulation in eudicots allowed a more relaxed or low purifying selection of the miRNA-responsive element region, which reflects its loss of functionality as a miR529 target. In line with this observation, the percentage of nucleotide identity within the “target site” block (including both miR156 and miR529 response elements) is lower among eudicot *SPL* genes (58.7 %) than among monocot and bryophyte ones (81.3 and 86.7 %, respectively) (Fig. 2). In monocots and *P. patens*, the “target site” block is more conserved, likely because it is functionally relevant as miR529 is still present in these species [24].

### Overexpression of a basal eudicot microRNA529 precursor in *A. thaliana* phenocopies miR156 overexpressor

Although miR156 is highly conserved, being present in all plant species assessed thus far, miR529 seems to be restricted to particular taxonomic groups [12]. To get a better view of the *MIR156/529* gene evolution, the phylogenetic relationship of these miRNAs was accessed using the maximum-likelihood (ML) approach. For phylogenetic analyses, we included *MIR156* and *MIR529* precursors from *Physcomitrella patens*, monocot species (*Oryza sativa*, *Zea mays*, *Brachypodium distachyon*, and *Sorghum bicolor*), a basal eudicot (*Aquilegia coerulea*), and precursors of *MIR156* genes from *Arabidopsis thaliana*. A consensus ML tree was generated in which two general, broad groups were readily observed (Additional file 3A). Group I comprised *MIR156* precursors from different species, while group II contained *MIR529* precursors from monocots and moss. Monocot *MIR529* precursors were grouped into a distinct subset of group II (Additional file 3A), suggesting that evolutionary divergence occurred in a common ancestor of land plants, which led to the split between *MIR529* genes of moss and flowering plants.

It had been proposed earlier that a key feature of miRNA evolution is that, once evolved, families and family members are rarely lost [2]. However, not all miRNAs are equally conserved and it has been recently shown that several miRNA losses occurred in families that evolved prior to the origin of spermatophytes [32]. Our data suggest that miR156 and miR529 families experienced dynamic duplications and losses across embryophytes, through which clade- or species-specific miRNA gene subgroups have arisen or were eliminated. For instance, *A. thaliana* has at least 10 *MIR156 loci* and 10 miR156-targeted *SPLs*, whereas rice has at least 12 *MIR156 loci*, two *MIR529* loci, and eight miR156-targeted and four miR156/miR529-targeted *SPLs* [28].

Interestingly, the predicted *MIR529* precursor from the basal eudicot *A. coerulea* [33] was grouped into group I, with *A. thaliana MIR156h* and *Aquilegia MIR156a* and *b* precursors, indicating a common origin of these miRNAs (Additional file 3A). Moreover, *Aquilegia MIR529* seems to be highly conserved with Arabidopsis *MIR156h* at both nucleotide and secondary structure levels (Additional file 3B). These observations raised the question of whether this *MIR* precursor of *Aquilegia* defined as *MIR529* is indeed a *MIR156* homolog. To test this hypothesis, we investigated whether *loci* flanking *Aquilegia* pre-miR529 are localized into syntenic blocks when comparing with monocot species. We firstly searched for such syntenic groups among distinct monocot species, including *Z. mays*, *O. sativa*, *B. distachyon*, and *S. bicolor* (Additional file 4A). The colinearity of genes around pre-miR529 locus is relatively conserved, therefore defining conserved syntenic block in monocots. Conversely, we did not detect any conservation of syntenic block including pre-miR529 from *Aquilegia*, although some orthologous genes were found to be present in this species (Additional file 4B). Moreover, there is no detectable synteny between *MIR156h*-associated regions from eudicots (*A. thaliana* and *V. vinifera*) and the *MIR529*-associated region from basal eudicot *A. coerulea* (Additional file 4B, C). This complete loss of synteny, along with our phylogenetic and sequence analyses, suggests that the basal eudicot *Aquilegia* lost a *bona-fide MIR529* gene, perhaps after the regulatory role of miR529 was perturbed due to mutations in the miR529 sequence as recently proposed [25].

**Fig. 3:**
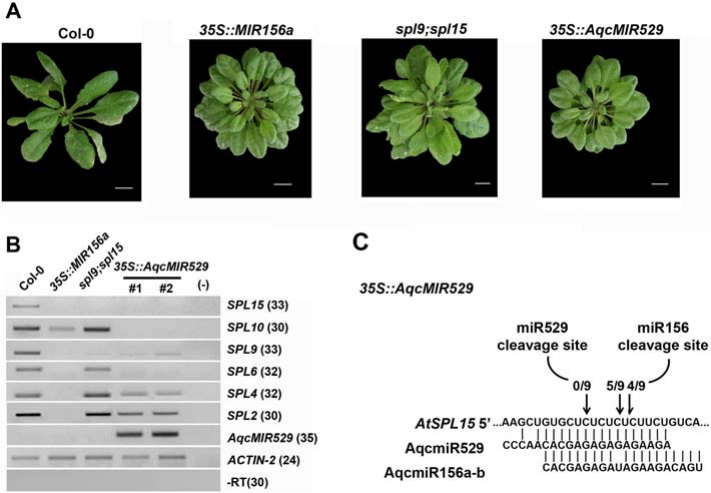
Phenotypic, expression, and RACE analyses of Arabidopsis *35S::AqcMIR529* plants. **a** Morphology of 25-day-old plants (Col-0 or wild type, *35S::MIR156a*, *spl9;spl15*, and *35S::AqcMIR529*). Scale bar represents 1 cm. **b** Stem–loop pulsed RT-PCR to detect *AqcMIR529* precursor and some *AtSPL* transcripts in Arabidopsis leaf tissues. Reactions without RT (−RT) and without cDNA (−) were used as negative controls. Numbers between brackets indicate PCR cycles. *Actin-2* was used as an internal control. **c** Modified 5′-RACE analyzes of cleaved SPL15 transcripts in *35S::AqcMIR529* leaf tissues. The 5′-ends of the *SPL15* cleavage products preferentially map to miR156

To determine whether *AqcMIR529* could be properly processed and could give rise to functional miRNAs, we constitutively expressed its foldback in *A. thaliana* plants under control of the viral 35S promoter, which confers strong and near-ubiquitous expression ([34]; such plants were hereafter referred to as *35S::AqcMIR529* lines; see Methods). We compared vegetative phenotypes among Col-0 or wild type, *35S:: AqcMIR529* lines, transgenic plants overexpressing the *AtMIR156a* precursor (*35S::AtMIR156a*; [35]), and the double mutant *spl9;spl15* (Fig. 3a). This mutant contains T-DNA insertions in both *AtSPL9* and *AtSPL15 loci*, which resulted in an accentuated reduction in their expression and function, although other *AtSPLs* are still functional. Transgenic *A. thaliana* plants transformed with the *Aquilegia*-overexpressing construct had phenotypes similar to *35S::MIR156a* plants, such as a high number of smaller, more rounded rosette leaves (Fig. 3a). Moreover, overexpression of the *MIR529* precursor from *Aguilegia* in *A. thaliana* led to the down-regulation not only of *AtSPL9* and *AtSPL15* genes (which contain both miR156 and miR529 response elements; Fig. 1), but also other *SPL* genes (Fig. 3b). Thus, *35S::AqcMIR529* lines showed a stronger tendency toward the phenotype of *35S::MIR156a* plants (Fig. 3a).

In line with the observed phenotypes and expression analyses, RACE analysis of *SPL15* cleavage sites demonstrated that transcripts are chiefly targeted by miR156 in *35S::AqcMIR529* plants (Fig. 3c). Based on these observations and given that *AqcMIR529* precursor is more conserved with *MIR156*-like precursors (Additional file 3), we propose at least two non-exclusive possibilities: (1) the predicted *AqcMIR529* precursor is more likely paralogous to *AqcMIR156a* and *b* genes and would produce a miRNA156-like small RNA; (2) *AqcMIR529* precursor might have accumulated mutations in the miR529 sequence as recently proposed [25], leading to a loss of miR529 biogenesis and/or function. As other basal eudicots seem to accumulate miR529-like small RNAs [36], we cannot rule out the possibility that miR529-like small RNAs still accumulate in specific *A. coerulea* tissues, since there is no available information regarding large-scale identification of small RNA populations in this basal eudicot.

### MiR156 and miR529 show overlapping expression patterns during rice vegetative development

**Fig. 4:**
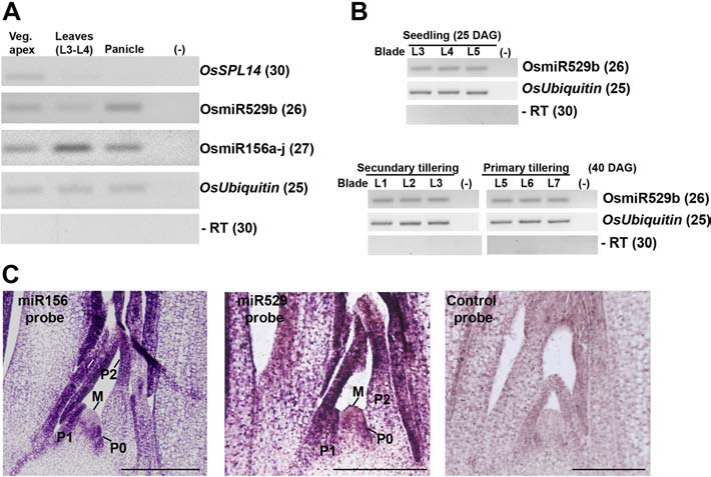
Expression patterns of miR529 and miR156 in *Oryza sativa*. **a** Stem–loop pulsed RT-PCR to detect OsmiR529b, OsmiR156a-j, and *OsSPL14* transcripts in vegetative apices (Veg. apex), leaf (L), and young panicle tissues. Reactions without RT (−RT) and without cDNA (−) were used as negative controls. Numbers between brackets indicate PCR cycles. DAG, days after germination. Rice *Ubiquitin* (LOC_Os03g0234200) was used as an internal control. **b** Stem–loop pulsed RT-PCR to detect OsmiR529b transcripts in leaf (L) tissues (Blade) from rice seedlings and tillering developmental stages. Reactions without RT (−RT) and without cDNA (−) were used as negative controls. Numbers between brackets indicate PCR cycles. Rice *Ubiquitin* (LOC_Os03g0234200) was used as an internal control. **c** Spatiotemporal expression patterns of miR529 and miR156 in rice shoot apical meristem (SAM). Probes of 3′-labelled LNA-modified oligonucleotides detecting miR156 and miR529 as described [38] were hybridized with longitudinal sections of the SAM from 25-DAG rice seedlings. A scramble-miRNA 3′-labelled LNA probe was used as a negative control. Purple staining shows probe localization. M, meristem; P, leaf primordia. Bars: 10 μm

Distinct from the basal eudicot *Aquilegia*, it is well documented that monocot species retained both *MIR156* and *MIR529* precursors in their genomes [12, 26, 37], raising the question of whether miR529 regulation has been retained in monocot species because it is essential or it is just a classical case of redundancy reflecting subfunctionalization in which miR529 has a limited effect as compared with miR156. Rice has two copies of *MIR529* precursors (*a* and *b*) in its genome. It has been shown that OsmiR529a* (or OsmiR529a-5p) is preferentially expressed in panicle, whereas OsmiR529b is ubiquitously expressed in roots, shoots, and panicle [26]. The authors, however, did not evaluate the expression of OsmiR156 or OsmiR529 in vegetative apices and young leaves, organs in which *SPLs* define an endogenous flowering pathway and control leaf maturation and initiation, respectively [19, 38]. To get more insights into the possible roles of OsmiR529 [26], we analyzed transcript accumulation patterns of OsmiR529b, OsmiR156a-j, and one of their common targets (*OsSPL14*) in vegetative apices, juvenile leaves (L3-L5), and young panicles. OsmiR529b and OsmiR156a-j were expressed in all tissues/organs evaluated, though at variable levels (Fig. 4a). OsmiR156a-j transcripts accumulated at high levels in juvenile leaf tissues, whereas *OsSPL14* was down-regulated in leaf and young panicle tissues (Fig. 4a). *OsSPL14* is targeted by both miRNAs in seedlings, whereas it seems to be predominantly targeted by miR529a-5p in panicle [26]. Likewise, OsmiR529b might also have a more prominent effect on the post-transcriptional regulation of *OsSPL14* expression in young panicle.

OsmiR156 is dynamically expressed during rice leaf development, and a gradual increase of OsmiR156 expression might be essential for regulating the temporal expression of target genes, including *OsSPL14* [38]. We evaluated the expression pattern of OsmiR529b in similar developed leaves in seedling (L3-L5) and tillering stages. In contrast with the observed OsmiR156 expression patterns [38], OsmiR529b was ubiquitously expressed in all leaf developmental stages (Fig. 4b), suggesting that this miRNA has a minor or negligible contribution for temporal control of the expression of *SPL* genes during rice leaf maturation. Nevertheless, it is also possible that miR529 function as a dampening miRNA to establish the correct balance of *SPL* targets during temporal leaf development in monocots.

Given that OsmiR529b and OsmiR156a-j transcripts accumulated in the vegetative apex (Fig. 4a), we decided to evaluate their spatial expression patterns in the shoot apical meristem (SAM) via in situ hybridization using specific probes as described ([38]; see Methods). Both miRNAs are expressed in incipient (P0) and developing leaf primordia, but not in the meristem proper (i.e., the peripheral and central zones) (Fig. 4c). Such expression pattern strengthened the data from Arabidopsis, which showed that miRNA regulation of *SPLs* occurs mainly in leaf primordia and that SPL activity may nonautonomously inhibit initiation of new leaves at the SAM, perhaps via auxin signaling pathways [39]. Our in situ data showed that OsmiR529a-b and OsmiR156a-j have overlapping spatial expression patterns in leaf primordia, which suggest that these miRNAs can redundantly regulate or collaborate to fine-tune regulation of target expression in these organs. However, based on reported higher levels of OsmiR156 expression compared with OsmiR529 expression [26], the contribution for *SPL* expression modulation is unlikely to be comparable for both miRNAs, mainly during early stages of rice vegetative development.

Modified 5′-RACE procedure can be used to access cleavage products of miRNA targets as well as the processing of miRNA precursors [40, 41]. Parallel analysis of RNA end (PARE) signatures that are derived from rice degradome and that only mapped to the pre-miRNAs can give additional evidences of roles of miR156 and miR529 in rice development. We therefore collected PARE signatures of *OsMIR156a-l* and *OsMIR529a-b* precursors from publicly available resources (see Methods). Based on available data, it seems that rice *MIR156* and *MIR529* precursors are differentially processed, which may lead to differential miRNA accumulation across rice tissues/organs (Fig. 4 and Additional file 5). Even within the *OsMIR156* family, specific members are differentially processed. For example, *OsMIR156k* and *–l* showed fewer PARE signatures when compared with the remaining *MIR156* precursors. Likewise, *OsMIR529a* and *b* precursors have a smaller amount of signatures when compared with most *OsMIR156* precursors (Additional file 5). It is possible that differential precursor processing also account for the distinct functions of OsmiR156 and OsmiR529 in development. It would be interesting in the future to address the question of whether miR529 regulation of *SPLs* is crucial for rice vegetative development. In contrast to miR156, the effects of over-expressing or down-regulating miR529 have yet to be examined in transgenic/mutant monocot plants.

### Conserved miR529-responsive element is functional in *AtSPL9* and *AtSPL15* genes

**Fig. 5:**
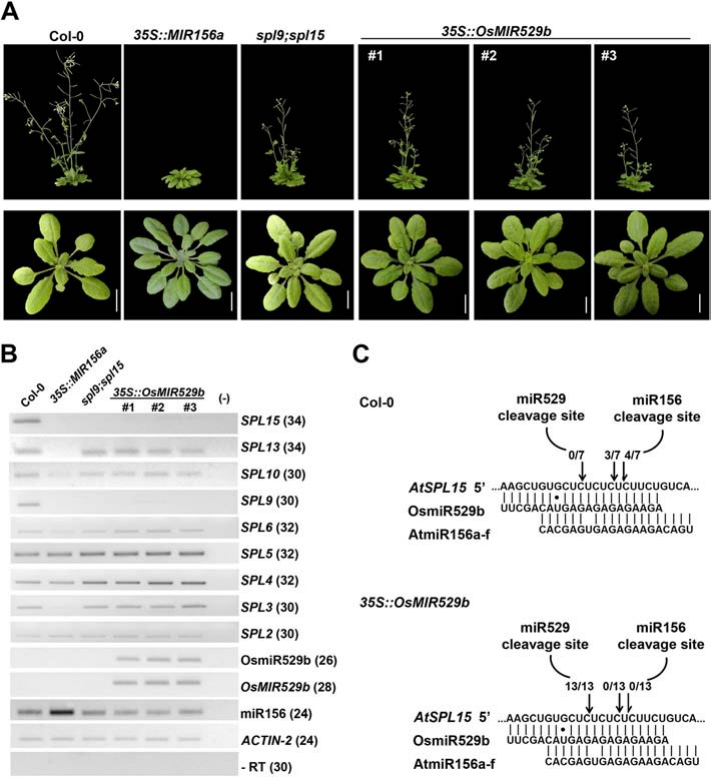
Phenotypic and expression analyses of *35S::OsMIR529b* Arabidopsis plants. **a** Morphology of six-week-old (*upper pannel*) and 25-day-old (*lowerpannel*) plants (Col-0 or wild type, *35S::MIR156a*, *spl9;spl15* mutant, *35S::OsMIR529b*). Scale bar represents 1 cm. **b** Stem–loop pulsed RT-PCR to detect *OsMIR529b* precursor, OsmiR529b, miR156, and *AtSPL* transcripts in Arabidopsis leaf tissues. Reactions without RT (−RT) and without cDNA (−) were used as negative controls. Numbers between brackets indicate PCR cycles. *Actin-2* was used as an internal control. **c** Modified 5′-RACE analyses of cleaved *SPL15* transcripts in Col-0 and *35S::OsMIR529b* leaf tissues. The 5′-ends of the *SPL15* cleavage products preferentially map to miR156 (in Col-0) and miR529b binding sites (in *35S::OsMIR529b*)

As discussed above, miR529 has been lost in eudicots like *A. thaliana*, yet the closely related *AtSPL9* and *AtSPL15* genes [29] harbor a conserved miR529-responsive element, similarly to their homolog in *A. lyrata* (Fig. 1c). Given that miRNA::target gene interactions have been comprehensively identified in *A. thaliana* [15, 17, 42], we asked whether miR529 binding site is functional in this core eudicot. To test this hypothesis, we generated transgenic Arabidopsis plants harboring the well-annotated rice *MIR529b* precursor under the control of viral 35S promoter (plants were hereafter referred to as *35S::OsMIR529b* lines). As *AtSPL9* and *AtSPL15* are involved in regulating leaf initiation and flowering time, we compared vegetative and flowering phenotypes among Col-0, *35S::OsMIR529b* lines, transgenic plants overexpressing the *AtMIR156a* precursor, and the double mutant *spl9;spl15* (Fig. 5a). At least three independent *35S::OsMIR529b* lines displayed similar phenotypes as the double mutant *spl9;spl15*, such as an increased number of rosette leaves in combination with a slight delay in flowering, albeit these phenotypes are less pronounced than those from *35S::AtMIR156a* plants (Fig. 5a).

To further confirm the phenotypic similarities between *35S::OsMIR529b* and *spl9;spl15* plants, we evaluated the average number of juvenile and rosette leaves (Table 1). Under our long-day (LD) growing conditions the *35S::MIR156a* line produced 2.8 times more juvenile leaves than Col-0 (wild type), similarly to data previously reported [17], whereas *35S::OsMIR529b* and *spl9;spl15* plants produced, on average, only 1.4 times more. Likewise, the production of rosette leaves of *35S::OsMIR529b* lines showed a stronger tendency toward the phenotype of the *spl9;spl15* mutant (Table 1).

**Table 1:** Phenotypic evaluation of *35S::OsMIR529b* lines in comparison with Col-0, *spl9;spl15*, and *35S::MIR156a* plants under LD growing conditions

	Juvenile leaves^1^	Rosette leaves (DAG)				Bolting (DAG)	Anthesis^2^ (DAG)
		15	20	25	30		
Col-0	6.8 ± 0.5^c^	7.7 ± 0.7^c^	8.8 ± 0.9^c^	13.3 ± 0.8^c^	14.3 ± 0.9^c^	16.5 ± 1.2^d^	23.6 ± 1.6^c^
35S::MIR156a	19.4 ± 1.8^a^	15.0 ± 0.9^a^	19.5 ± 1.6^a^	35.6 ± 4.6^a^	52.9 ± 2.6^a^	45.0 ± 1.8^a^	54.0 ± 3.3^a^
spl9;spl15	10.3 ± 0.7^b^	11.0 ± 1.2^b^	13.3 ± 1.8^b^	21.1 ± 2.3^b^	29.4 ± 2.2^b^	20.0 ± 0.7^bc^	25.8 ± 1.9^b^
35S::OsMIR529b #1	10.3 ± 0.5^b^	12.3 ± 1.1^b^	13.0 ± 0.6^b^	20.3 ± 1.1^b^	26.8 ± 2.0^b^	19.4 ± 0.7^c^	24.8 ± 1.5^bc^
35S::OsMIR529b #2	10.0 ± 1.1^b^	11.4 ± 1.0^b^	12.6 ± 1.1^b^	20.3 ± 2.7^b^	27.5 ± 1.6^b^	18.9 ± 0.9^c^	23.4 ± 1.3^c^
35S::OsMIR529b #3	9.8 ± 0.7^b^	11.0 ± 1.3^b^	13.2 ± 0.9^b^	19.6 ± 1.7^b^	26.5 ± 1.9^b^	20.9 ± 1.2^b^	26.8 ± 2.2^b^

Fifteen plants per genotype were used for determination. DAG, days after germination. Average values followed by the same letter do not differ statistically (*P* < 0.01, Student’s t-test)

^1^Number of rosette leaves formed before the appearance of the first leaf with abaxial trichomes

^2^Opening of the first flower

In addition to estimating the number of leaves formed before the appearance of the first flowers, we also determined the time that *35S::OsMIR529b* lines needed to bolt as well as to anthesis (Table 1). On average, transgenic plants overexpressing OsmiR529b showed a slight delay in the transition to flowering (3.3 days) when compared with Col-0 (wild type). In line with the observations of Schwarz and co-workers [29], we also observed that the double mutant *spl9;spl15* showed an intermediate behavior between Col-0 and miR156 overexpressor (Table 1). Importantly, *spl9;spl15* plants did not differ statistically from *35S::OsMIR529b* lines in terms of transition to flowering and leaf development (Table 1). These data reinforced the observation that *OsMIR529b* overexpressors display similar vegetative and reproductive phenotypes as *spl9;spl15* mutant, likely due to the low levels of *SPL9* and *SPL15* transcripts in both genotypes.

MiR156 targets, besides *SPL9* and *SPL15*, exclusively other eight *SPL* family members [15] and these were shown to be down-regulated in *AtMIR156b*-overexpressing plants [17]. In comparison with *spl9;spl15* double mutant and *35S::OsMIR529b* lines, *35S::MIR156a* line displays more severe, aberrant vegetative and reproductive phenotypes (Table 1; [35]), which is likely due to the fact that additional miR156-targeted *SPL* genes act redundantly to regulate leaf initiation and phase change [43]. Conversely, as in the *spl9;spl15* double mutant, only *AtSPL9* and *AtSPL15* genes may be repressed in *35S::OsMIR529b* lines, rendering them a less aberrant phenotype (Fig. 5a). To confirm this hypothesis, we evaluated the expression patterns of several *SPL* family members in leaf tissues of *35S::OsMIR529b* lines, *spl9;spl15* mutant, *35S::MIR156a* line, and Col-0. We also evaluated the presence of transcripts from *OsMIR529b* precursor and the accumulation of its respective mature miRNA (Fig. 5b). As expected, several *SPL* genes were down-regulated in the *35S::MIR156a* plants (Fig. 5b). However, in line with our phylogenetic (Fig. 1b) and phenotypic data (Table 1), only transcript levels of *AtSPL9* and *AtSPL15* showed a severe reduction in *35S::OsMIR529b* plants. Moreover, levels of miR156 transcripts were similar in Col-0 and *35S::OsMIR529b* plants (Fig. 5b), suggesting that the accentuated reduction in *SPL9* and *SPL15* expression in *OsMIR529b* overexpressors is most likely due to the accumulation of rice miR529b transcripts. Together, these results substantiated the fact that monocot *MIR529* precursor is correctly processed in a core eudicot to generate mature miR529 transcripts and down-regulate specific *SPL* genes.

To confirm the post-transcriptional regulation of *AtSPL* genes by miR529b, we mapped cleavage sites in *SPL15* transcripts in *35S::OsMIR529b* plants employing the modified 5′-RACE approach [40]. RACE analyses showed that cleavage sites occurred between the base 10 and 11 of the miR529b (Fig. 5c), similarly to what has been described for monocots [44]. These results robustly showed that *SPL15* is mainly regulated by OsmiR529b in Arabidopsis *35S::OsMIR529b* plants, demonstrating a conserved function of miR529 in post-transcriptionally regulating specific *SPL* family members. Importantly, our data imply that the miR529-responsive element is conserved and functional in Arabidopsis *SPL9* and *SPL15* genes, likely due to the selective constraint on the amino acid or RNA secondary structure of the region surrounding miR156/529-responsive element.

## Conclusions

We have shown that, although *MIR529* genes have been lost in Arabidopsis and perhaps in all eudicot species, particular *SPL* genes in these species retained the miR529-responsive element, possibly due to the maintenance of synonymous codons for efficient miR156 binding and proper function [10]. More specifically, *A. thaliana SPL9* and *SPL15* genes retained a functional miR529-responsive element, even in the absence of a miR529-generated locus. Similarly to monocot *SPLs*, eudicot *SPL* genes containing the miR156/529-responsive element appear to be under evolutionary constraints distinct from those containing only the miR156-responsive element. Such tendency would be indicative of target evolution constrained by miRNA-mediated regulation.

It is possible that the interplay between miR156 and miR529 regulation of specific *SPLs* be important to fine-tune flower architecture development in monocots, particularly in grasses [26, 37]. As Arabidopsis does not have miR529, perhaps particular miR156 family members (such as miR156h.2, which is preferentially expressed in flower tissues; [45]) functionally replace miR529. It is conceivable that other core rosids and/or closely related species of *A. thaliana* share similar miR156/miR529/SPL evolutionary history, though such confirmation requires future studies.

## Methods

### Plant material and growth conditions

*Arabidopsis thaliana* plants (ecotype Columbia-0 or Col-0) were grown at 21 °C (day)/19 °C (night) under long-day conditions (16 h light/8 h dark). Transgenic plants *35S::MIR156a* and the double mutant *spl9-1;spl15-2* were described [35]. Transgenic plants were confirmed by PCR genotyping.

For transgenic Arabidopsis plants, the binary constructs *35S::OsMIR529b* and *35S::AqcMIR529* were delivered into *Agrobacterium tumefaciens* GV3101 (pMP90) by the electroporation method. Transgenic plants were generated by the floral dipping method [46] and screened with 50 mg/mL kanamycin on half-strength MS plates. At least six independent kanamycin-resistant lines were selected for transgene integration by PCR and subsequently examined for transgene expression levels (data not shown). Further analyses were performed with selected lines in the T3 generation.

Rice seeds (*Oryza sativa* ssp *japonica*) were germinated on soil, and plants were grown under greenhouse conditions.

### DNA constructs

Oligonucleotide primers for all constructs are given in the Additional file 6. A 1000-bp fragment encompassing the *OsMIR529b* precursor was amplified from genomic DNA of *O. sativa*. The PCR product was subcloned into pGEM (promega) and sequenced. The confirmed *OsMIR529b* precursor was digested with *BamHI* and *SacI* restriction enzymes and subsequently cloned into the binary vector pBI121 behind the CaMV35S promoter. For *35S::AqcMIR529* construction, a 125-bp fragment encompassing the annotated *AqcMIR529* precursor [33] was amplified from genomic DNA of *A. coerulea*, sequenced, and further cloned into the plant binary destination vector pK7WG2 (Gateway System; [47]) behind the CaMV35S promoter.

### RNA extraction and stem–loop pulsed reverse transcriptase (RT)-PCR

Total RNA from Arabidopsis (leaf tissues) and rice (vegetative apices, leaf, and panicle tissues) was extracted using Trizol reagent (Life Technologies, USA) according to manufacturer’s instructions and subsequently treated with DNAse I (Life Technologies, USA). For miRNA and mRNA detection, DNAse I-treated RNA (2.0 μg) was reverse-transcribed to generate the first-strand cDNA, according to Varkonyi-Gasic et al. [48]. Oligo(dT) primer was also added to the reaction for detecting target mRNAs and internal controls. cDNA dilutions were used for PCR reactions as follows: 1.0 μL cDNA, 1.5 mM Magnesium Sulfate, 0.25 mM each dNTP, 10 pmol each primer, and 1 U of Taq DNA Polymerase (Promega, USA). The reactions were done under the following conditions: 94 °C for two minutes and appropriate cycle numbers of 94 °C for 20 s, 60 °C for 30 s, and 72 °C for 45 s. All reactions were repeated at least twice with two biological samples. Primer sequences are described in Additional file 6.

### Analysis of 5′-RACE

Five micrograms of total RNA from rosette leaves of Arabidopsis plants (Col-0, *35S::OsMIR529b* and *35S::AqcMIR529*) was ligated to a RNA adapter, in a reaction mixture containing 0.5 U/μL of T4 RNA Ligase, 4 U/μL RNAse inhibitor, and 1 mM ATP. The subsequent steps were performed according to the manufacturer’s guide of the GeneRacer kit (Invitrogen). The first PCR was done using the following *AtSPL15* specific primer: 5′-AGCCATTGTAACCTTATCGGAGAATGAG. The PCR reaction was subsequently used as a template for a semi-NESTED PCR with an internal *AtSPL15*-specific primer (5′-TCATCGAGTCGAAACCAGAAGAT). After amplification, 5′-RACE products were gel-purified and cloned, and at least eight independent clones were randomly chosen and sequenced.

### Phenotypic analysis

The number of rosette leaves was measured during several developmental stages (15, 20, 25, and 30 days after germination or DAG). Flowering and bolting time as well as the number of juvenile leaves were estimated as described. Data were subjected to statistical analyses by using the program ASSISTAT version 7.6 beta (t-student *P* <0.01).

### In situ hybridization

Non-radioactive in situ hybridization was done as described [49]. *Oryza sativa* vegetative apices were collected from seedlings 25 days after germination. Locked nucleic acid probes with sequences complementary to miR56 and miR529 as described [38] and negative control Scramble-miR (5′-GTGTAACACGTCTATACGCCCA) were synthesized by Exiqon (USA) and digoxigenin-labeled with the DIG Oligonucleotide 3′-end Labeling kit (Roche Applied Science, USA). Ten picomoles of each probe was used for each slide. Hybridization and washing steps were performed at 55 °C.

### Phylogenetic and sequence analysis

Sequences of *MIR156* and *MIR529* precursors (pre-miRNAs) were retrieved from miRBase v.21 (http://www.mirbase.org/) whereas SPL sequences were retrieved from PHYTOZOME v. 9.1 (http://www.phytozome.net/) and TAIR (https://www.arabidopsis.org/). For miRNA precursors, retrieved sequences were aligned using ClustalW [50] using default values. Phylogenetic analyses were performed in MEGA v. 5.05 [51] using default values. Phylogenetic inference was done using maximum-likelihood (ML) method with Bootstrap analysis (1000 trees).

The DNA sequences of *SPLs* were aligned using Muscle algorithm [52] and the well-aligned blocks were selected using Gblocks server (http://molevol.cmima.csic.es/castresana/Gblocks_server.html) by the most stringent option. Multiple sequence alignment is depicted in Additional file 7. The alignment was submitted to the estimation of proportion of invariant sites and substitution saturation test using the algorithm of Xia test implemented in DAMBE5 software [53]. The option for the best-fit evolutionary model was performed using Akaike information criterion implemented in jModelTest [54]. The phylogenetic reconstruction was determined by ML and Bayesian inference methods, using PhyML v3.0 [55] and Beast v1.8.0 [56], respectively, the latter being implemented in CIPRES Science Gateway (https://www.phylo.org/). The approximate likelihood ratio test or aLRT [57] was used for ML analysis. The posterior probability estimates were calculated for Bayesian inference. The software Tracer was applied to determine the burn-in (using the log likelihood scores) in Bayesian method generation and the TreeAnnotator [54] to summarize the data after the exclusion of the trees that appeared outside the convergence area. The proportion of invariable sites and gamma distribution (number of categories = 4) was estimated and random local clock model for Bayesian analysis was also used.

PARE signatures mapping to *OsMIR156* and *OsMIR529* precursors and RNA-seq and sRNA signatures were retrieved from Rice Next-Gen sequence DBs (http://mpss.udel.edu/). Sequence abundance was estimated by normalizing all samples to TP10M (transcript per 10 million reads).

### Nonsynonymous (Ka) and synonymous (Ks) substitution calculation

The selective pressure analysis (Ka/Ks) was performed using best-aligned blocks for nucleotide sequences encompassing the SBP domain plus few nucleotides upstream/downstream (named “SBP domain”). Eudicot, monocot, and bryophyte SPL sequences were carefully selected based on the phylogenetic tree depicted in Fig. 1b and phylogenetic analyses reported previously [29]. Codons of each DNA sequence for each edited alignment were selected using an in-house Python script. Values of Ka/Ks were estimated by comparing sequences among and within eudicot, monocot, and bryophyte groups through the software KaKs_Calculator using the model selection (MS) method [58]. Selected “target site” sequences were aligned using the Muscle algorithm, and Logos were generated using Geneious tools (http://www.geneious.com).

### Synteny analysis

Based on coordinates of neighbor genes of sites of pre-miR529 *in O. sativa* (*OsMIR529a* and *OsMIR529b*), *A. coerulea*, and pre-miR156h from *V. vinifera* and *A. thaliana*, the conservation of syntenic blocks among and within monocot and eudicot species was searched in Genomicus Plants v.16.03 [59]. For syntenic mapping of *Aquilegia coerulea*, we firstly used coordinates of pre-miR529 sites (scaffold_4:4,760,784..4,810,783) from Phytozome database to map flanking genes around *aqc-MIR529* locus. Orthologous genes for those loci in selected eudicots and monocots were queried in Genomicus Plants v.16.03 [59]. Phytozome database was also used to search for homologs in *A. coerulea* of pre-miR529 and pre-miR156h neighbor genes from *O. sativa*, *V. vinifera*, and *A. thaliana*, respectively.

## Availability of supporting data

All published datasets referred to in the manuscript are cited in the reference list. All the supporting data are included as additional files.

## Accession numbers

AGI identifiers for *Arabidopsis thaliana* genes are as follows: *SPL2*, At5g43270; *SPL3*, At2g33810; *SPL4*, At1g53160; *SPL5*, At3g15270; *SPL6*, At1g69170; *SPL9*, At2g42200; *SPL10*, At1g27370; *SPL13*, At5g50570; *SPL15*, At3g57920; *Actin-2*, At3g18780.

## Additional files

Additional file 1:
**Predicted** ***SBP/SPL*** **targets from eudicot, monocot, and bryophyte species that contain miR156/529-responsive element.** (PDF 111 kb) Additional file 2:
**Estimated Ka/Ks ratios for** ***SPL*** **genes from distinct species.** (XLS 33 kb) Additional file 3:
**Unrooted phylogenetic tree and sequence alignments depict the relationship between** ***MIR156*** **and** ***MIR529*** **precursors from representative species.** (A) Available *MIR156* and *MIR529* precursor sequences were retrieved from miRbase v. 21 (http://www.mirbase.org/). Phylogenetic analysis was performed using maximum-likelihood with bootstrap analysis (1000 trees). The percentage of trees (above 50 %) in which the associated taxa clustered together is shown next to the branching sites. ath, *Arabidopsis thaliana*; aqc, *Aquilegia coerulea*; bdi, *Brachypodium distachyon*; osa, *Oryza sativa*; ppt, *Physcomitrella patens*; sbi, *Sorghum bicolor*; zma, *Zea mays*. (B) Sequence alignments of *MIR529* and *MIR156* precursors. Al, *Arabidopsis lyrata*. Alignments were done using ClustalW [50]. Harpin structure predictions of *MIR529* and *MIR156* precursors were estimated using MFOLD3.2 algorithm [60]. (PDF 569 kb) Additional file 4:
**Conservation of syntenic blocks.** (A) Genomic regions containing homologs of rice pre-miR529a/b neighbor genes. (B) Genomic regions containing homologs of *Aquilegia* pre-miR529 neighbor genes. (C) Genomic regions containing homologs of Arabidopsis and grape pre-miR156h neighbor genes. Arrows sharing the same color in different species designate orthologous genes. Arrows with gray and black lines indicate paralogs if they share the same color, although not all black lined arrows indicated orthologs (please see Genomicus manual; http://www.dyogen.ens.fr/genomicus). The descriptions on top of some arrows indicate locus name and position in that particular genome; *, regions of pre-miRNAs; “:”, loci not shown. The quantity of this symbol indicates the number of loci; “//”, large genomic blocks not shown. Collinear arrows surrounded by a box indicate conserved syntenic blocks. (PDF 458 kb) Additional file 5:
**PARE and** **RNA-seq** **data for rice** ***MIR156*** **and** ***MIR529*** **precursors.** (PDF 58 kb) Additional file 6:
**Oligonucleotide sequences used in this work.** (PDF 84 kb) Additional file 7:
**Multiple sequence alignment of selected SPL nucleotide sequences.** (PDF 86 kb)
