# Erratum to: Functional and evolutionary analyses of the miR156 and miR529 families in land plants

**DOI:** 10.1186/s12870-016-0802-8

**Published:** 2016-07-07

**Authors:** Edna Gicela Ortiz Morea, Eder Marques da Silva, Geraldo Felipe Ferreira e Silva, Guilherme Targino Valente, Carlos Hernan Barrera Rojas, Michel Vincentz, Fabio Tebaldi Silveira Nogueira

**Affiliations:** Laboratório de Genetica Molecular do Desenvolvimento Vegetal, Departamento de Ciencias Biológicas, Escola Superior de Agricultura ‘Luiz de Queiroz’, Universidade de Sao Paulo, Avenida Padua Dias, 11, 13418-900 Piracicaba, Sao Paulo Brazil; Departamento de Genética, Instituto de Biociencias, Universidade Estadual Paulista (UNESP), Botucatu, Sao Paulo Brazil; Departmento de Bioprocessos e Biotecnologia, Universidade Estadual Paulista (UNESP), Botucatu, Sao Paulo Brazil; Centro de Biologia Molecular e Engenharia Genetica (CBMEG), Universidade Estadual de Campinas, Campinas, Sao Paulo Brazil

## Erratum

Unfortunately, the original version of this article [1] contained an error. The spelling of the author “Fabio Tebaldi Silveira Nogueira” was misspelt “Fabio Tebaldi Siveira Nogueira”. This has been corrected in the original article and can also be seen correctly in the author list above.
