# Supplementary material for: Functional and evolutionary analyses of the miR156 and miR529 families in land plants
Source: BMC Plant Biol. 2016 Feb 3;16:40. doi: 10.1186/s12870-016-0716-5 (PMC4739381; doi:10.1186/s12870-016-0716-5)
Supplement: Additional file 1: — Predicted SBP/SPL targets from eudicot, monocot, and bryophyte species that contain miR156/529-responsive element. (PDF 111 kb) [file 12870_2016_716_MOESM1_ESM.pdf]

Additional file 1. Predicted *SBP/SPL* targets from eudicot, monocot, and bryophyte species that contain miR156/529-responsive element.

Family/Class	SBP/SPL[a]	ID	Position	No. Mismatches[b]	Target Sequence (5´-3')[c]
Brassicaceae/Eudicot	*AtSPL15*	At3g57920	CDS	1	AAGCU**GUGCUCUCUCUCUUCU**GUCA
	AtSPL9	At2g42200	CDS	1	AAACU**GUGCUCUCUCUCUUCU**GUCA
	AlSPL15	ARALYDRAFT_486243	CDS	1	AAGCU**GUGCUCUCUCUCUUCU**GUCA
	ThSPL	AK353538	CDS	2	AAACU**GUGCUCUCUCUCUUCU**GUCA
Vitaceae/Eudicot	*VvSPL9*	NM_001280969.1	CDS	1	AAGCU**GUGCUCUCUCUCUUCU**GUCA
Solanaceae/Eudicot	*SlSBP15*	Solyc10g078700	CDS	1	AAGUG**GUGCUCUCUCUCUUCU**GUCA
	SlSBP6a	Solyc03g114850	CDS	3	CAGCA**AUGCUCUCUCUCUUCU**GUCA
	SlSBP6c	Solyc12g038520	CDS	1	UGGCU**GUGCUCUCUCUCUUCU**GUCA
Rosaceae/Eudicot	*MdSPL1*	HM122684	CDS	1	AAGCU**GUGCUCUCUCUCUUCU**GUCA
	FaSPL9	JN979457	CDS	3	GAGUU**GUGCUCUCUCUCUUCU**GUCA
Salicaceae/Eudicot	*PtSPL1*	POPTR_0016s04890g	CDS	2	GAGCU**GUGCUCUCUCUCUUCU**GUCA
	PtSPL2	POPTR_0010s16400g	CDS	2	UAGCU**GUGCUCUCUCUCUUCU**GUCA
Fabaceae/Eudicot	*GmSPL9*	LOC100789301	CDS	2	AAACU**GUGCUCUCUCUCUUCU**GUCA
	MtSPL	MTR_7g092930	CDS	2	AAACU**GUGCUCUCUCUCUUCU**GUCA
Rutaceae/Eudicot	*PontSPL9*	FJ502237	CDS	2	AAACU**GUGCUCUCUCUCUUCU**GUCA
Plantaginaceae/Eudicot	*AmSPL4*	AJ011622	CDS	3	CAGCG**GUGCUCUCUCUCUUCU**GUCA
Euphorbiaceae/Eudicot	*RcLIGULELESS1*	XM_002532297	CDS	3	AUCCU**GUGCUCUCUCUCUUCU**GUCA
	RcSPL	XM_002512008	CDS	3	CAGUU**GUGCUCUCUCUCUUCU**GUCA

Additional Table 1. Continued

Family/Class	*SBP/SPL* gene	ID	Position	No. Mismatches	Target Sequence (5´-3’)
Panicoideae/Monocot	*ZmSBP1*	GRMZM2G126018	CDS	2	CAGCU**GUGCUCUCUCUCUUCU**GUCA
	ZmSBP5	GRMZM2G061734	CDS	2	GGAUU**GUGCUCUCUCUCUUCU**GUCA
	TSH4	GRMZM2G307588	CDS	2	CAGCU**GUGCUCUCUCUCUUCU**GUCA
	ZmSBP7	GRMZM2G460544	CDS	2	CAGCU**GUGCUCUCUCUCUUCU**GUCA
	SbSPL2	SORBIDRAFT_03g044160	CDS	3	CAACU**GUGCUCUCUCUCUUCU**GUCA
	SbSPL5	SORBIDRAFT_07g027740	CDS	3	UAGCU**GUGCUCUCUCUCUUCU**GUCA
Ehrhartoideae/Monocot	*OsSPL2*	LOC_Os01g69830	CDS	3	CAACU**GUGCUCUCUCUCUUCU**GUCA
	OsSPL14	LOC_Os08g39890	CDS	2	AAGCU**GUGCUCUCUCUCUUCU**GUCA
	OsSPL17	LOC_Os09g31438	CDS	2	CAGCU**GUGCUCUCUCUCUUCU**GUCA
Pooideae/Monocot	*BdSPL14*	LOC100836973	CDS	2	CAGCU**GUGCUCUCUCUCUUCU**GUCA
	BdSPL17	LOC100824643	CDS	2	CAGCU**GUGCUCUCUCUCUUCU**GUCA
Funariaceae/Bryophyta	*PpSPL3*	Pp1s194_53V6	CDS	2	UGGCC**GUGCUCUCUCUCUUCU**GUCA
	PpSPL6	Pp1s50_125V6	CDS	2	UGGUC**GUGCUCUCUCUCUUCU**GUCA
	PpSPL13	Pp1s194_57V6	CDS	2	UGGCC**GUGCUCUCUCUCUUCU**GUCA

[a]miR156/529 site-containing *SBP/SPL* genes identified in each species. *At*, *Arabidopsis thaliana*; *Al*, *Arabidopsis lyrata*; *Th*, *Thellungiella halophila*; *Vv*, *Vitis vinifera*; *Sl*, *Solanum lycopersium*; *Md*, *Malus domestica*; *Fa*, *Fragaria ananassa*; *Pt*, *Populus trichocarpa*; *Gm*, *Glycine max*; *Mt*, *Medicago truncatula*; *Pont*, *Poncirus trifoliata*; *Am*, *Antirrhinum majus*; *Rc*, *Ricinus communis*; *Zm*, *Zea mays*; *Os*, *Oryza sativa*; *Bd*, *Brachypodium distachyon; Aqc*, *Aquilegia caerulea*; *Pp*, *Physcomitrella patens*.

[b]Number of mismatches between miR529 and targets.

[c]Target sequences containing response elements for both miR156 and miR529. The first five underlined nucleotide acids indicate target sequence for miR529 and the last four show target sequence for miR156. The **bold** segment indicates the overlapped target sequence for miR156 and miR529.
