# Supplementary material for: Functional and evolutionary analyses of the miR156 and miR529 families in land plants
Source: BMC Plant Biol. 2016 Feb 3;16:40. doi: 10.1186/s12870-016-0716-5 (PMC4739381; doi:10.1186/s12870-016-0716-5)
Supplement: Additional file 3: — Unrooted phylogenetic tree and sequence alignments depict the relationship between MIR156 and MIR529 precursors from representative species. (A) Available MIR156 and MIR529 precursor sequences were retrieved from miRbase v. 21 (http://www.mirbase.org/). Phylogenetic analysis was performed using maximum-likelihood with bootstrap analysis (1000 trees). The percentage of trees (above 50 %) in which the associated taxa clustered together is shown next to the branching sites. ath, Arabidopsis thaliana; aqc, Aquilegia coerulea; bdi, Brachypodium distachyon; osa, Oryza sativa; ppt, Physcomitrella patens; sbi, Sorghum bicolor; zma, Zea mays. (B) Sequence alignments of MIR529 and MIR156 precursors. Al, Arabidopsis lyrata. Alignments were done using ClustalW [50]. Harpin structure predictions of MIR529 and MIR156 precursors were estimated using MFOLD3.2 algorithm [60]. (PDF 569 kb) [file 12870_2016_716_MOESM3_ESM.pdf]

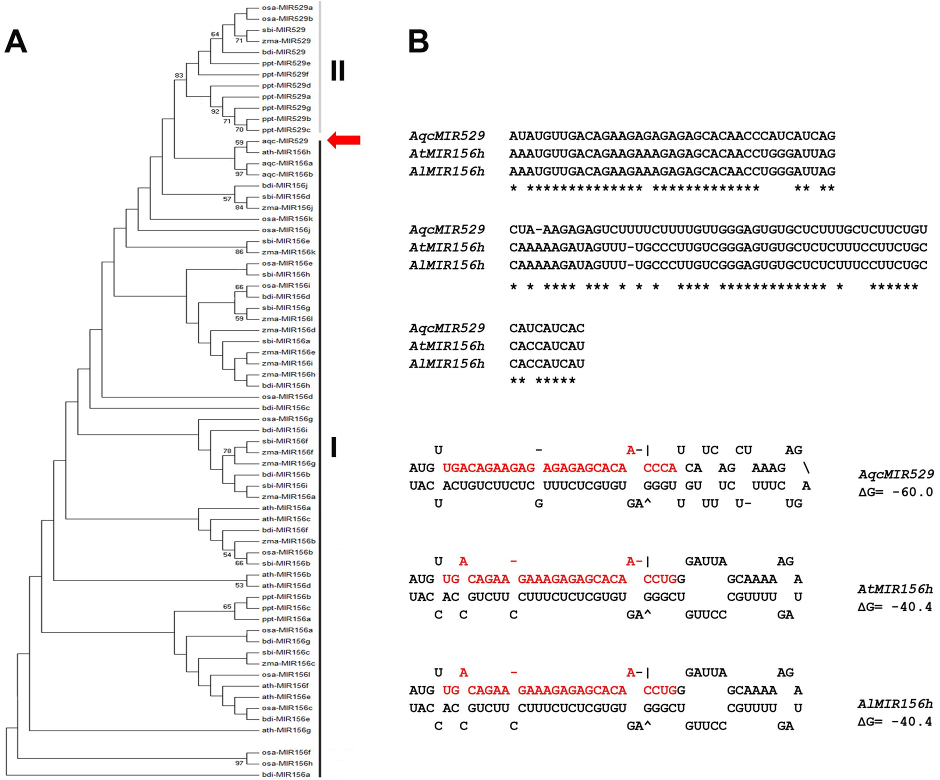
A
osa-MIR529a
osa-MIR529b
sbi-MIR529
zma-MIR529
bdi-MIR529
ppt-MIR529e
ppt-MIR529f
ppt-MIR529d
ppt-MIR529a
ppt-MIR529g
ppt-MIR529b
ppt-MIR529c
aqc-MIR529
ath-MIR156h
aqc-MIR156a
aqc-MIR156b
bdi-MIR156j
sbi-MIR156d
zma-MIR156j
osa-MIR156k
osa-MIR156j
sbi-MIR156e
zma-MIR156k
osa-MIR156e
sbi-MIR156h
osa-MIR156i
bdi-MIR156d
sbi-MIR156g
zma-MIR156l
zma-MIR156d
sbi-MIR156a
zma-MIR156e
zma-MIR156i
zma-MIR156h
bdi-MIR156h
osa-MIR156d
bdi-MIR156c
osa-MIR156g
bdi-MIR156i
sbi-MIR156f
zma-MIR156f
zma-MIR156g
bdi-MIR156b
sbi-MIR156i
zma-MIR156a
ath-MIR156a
ath-MIR156c
bdi-MIR156f
zma-MIR156b
osa-MIR156b
sbi-MIR156b
ath-MIR156b
ath-MIR156d
ppt-MIR156b
ppt-MIR156c
ppt-MIR156a
osa-MIR156a
bdi-MIR156g
sbi-MIR156c
zma-MIR156c
osa-MIR156l
ath-MIR156f
ath-MIR156e
osa-MIR156c
bdi-MIR156e
ath-MIR156g
osa-MIR156f
osa-MIR156h
bdi-MIR156a
II
I
B
AqcMIR529 AUAUGUUGACAGAAGAGAGAGAGCACAACCCAUCAUCAG
AtMIR156h AAAUGUUGACAGAAGAAAGAGAGCACAACCUGGGAUUAG
AlMIR156h AAAUGUUGACAGAAGAAAGAGAGCACAACCUGGGAUUAG
AqcMIR529 CUA-AAGAGAGUCUUUUCUUUUGUUGGGAGUGUGCUCUUUGCUCUUCUGU
AtMIR156h CAAAAAGAUAGUUU-UGCCCUUGUCGGGAGUGUGCUCUCUUUCCUUCUGC
AlMIR156h CAAAAAGAUAGUUU-UGCCCUUGUCGGGAGUGUGCUCUCUUUCCUUCUGC
AqcMIR529 CAUCAUCAC
AtMIR156h CACCAUCAU
AlMIR156h CACCAUCAU
AqcMIR529
ΔG= -60.0
AtMIR156h
ΔG= -40.4
AlMIR156h
ΔG= -40.4
