# Supplementary figures and images for: Functional and evolutionary analyses of the miR156 and miR529 families in land plants
Source: BMC Plant Biol. 2016 Feb 3;16:40. doi: 10.1186/s12870-016-0716-5 (PMC4739381; doi:10.1186/s12870-016-0716-5)

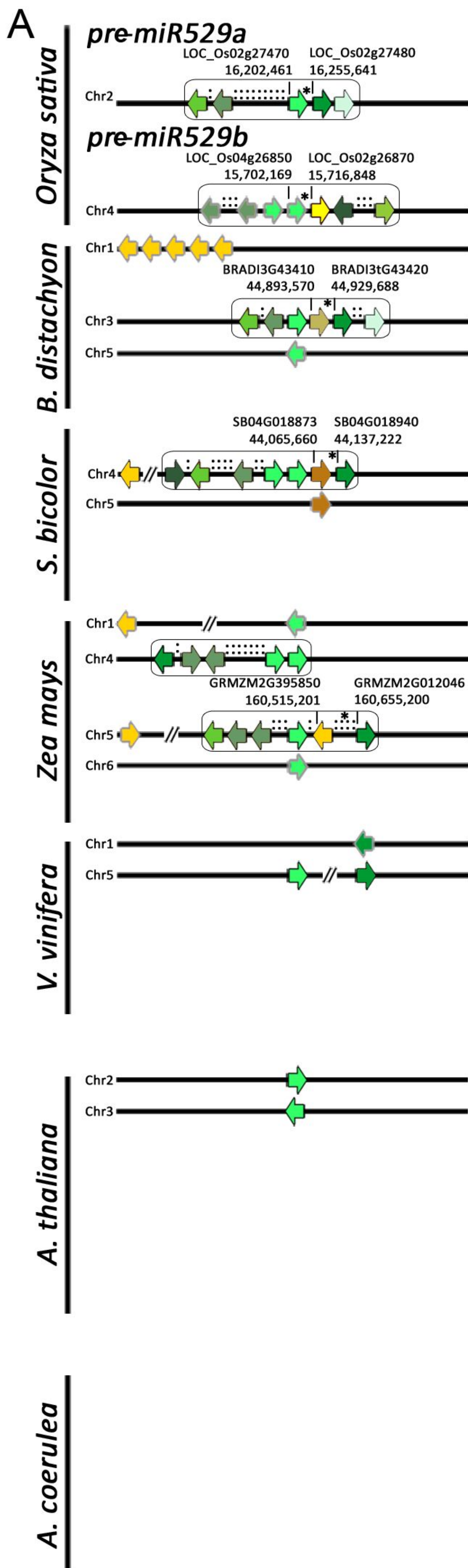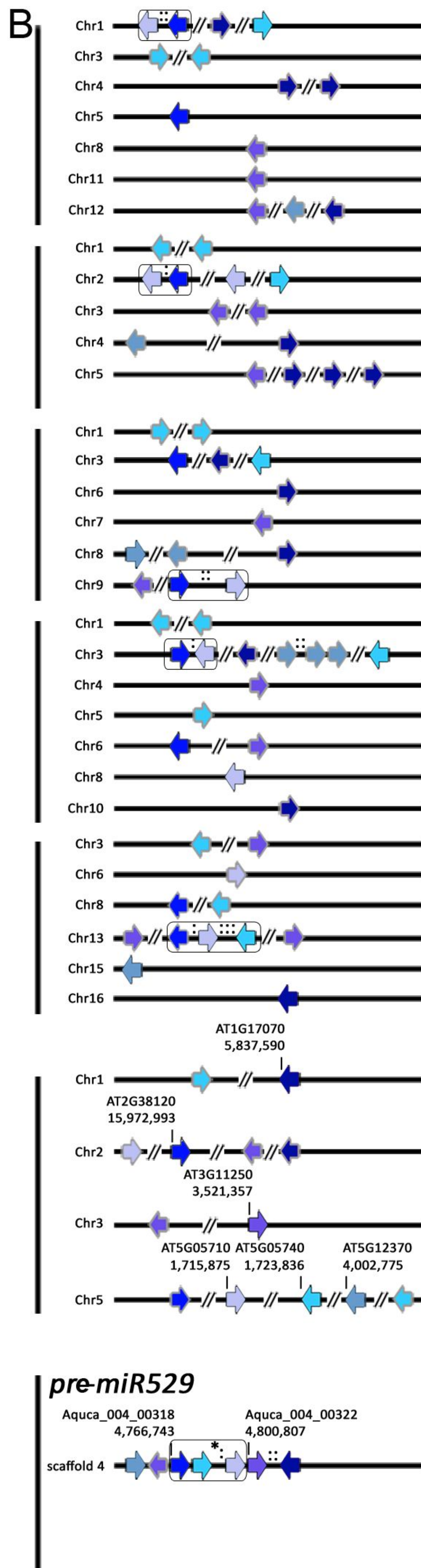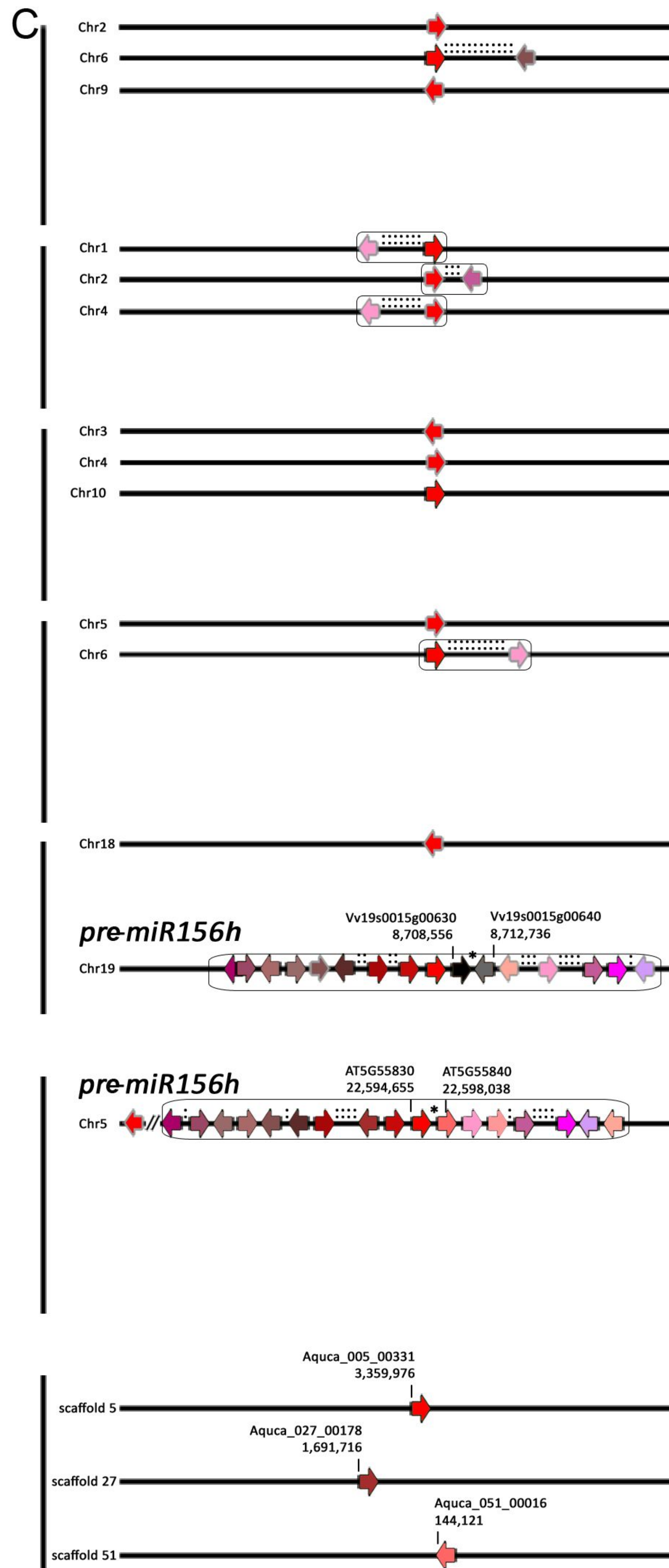

Supplement: Additional file 4: — Conservation of syntenic blocks. (A) Genomic regions containing homologs of rice pre-miR529a/b neighbor genes. (B) Genomic regions containing homologs of Aquilegia pre-miR529 neighbor genes. (C) Genomic regions containing homologs of Arabidopsis and grape pre-miR156h neighbor genes. Arrows sharing the same color in different species designate orthologous genes. Arrows with gray and black lines indicate paralogs if they share the same color, although not all black lined arrows indicated orthologs (please see Genomicus manual; http://www.dyogen.ens.fr/genomicus). The descriptions on top of some arrows indicate locus name and position in that particular genome; *, regions of pre-miRNAs; “:”, loci not shown. The quantity of this symbol indicates the number of loci; “//”, large genomic blocks not shown. Collinear arrows surrounded by a box indicate conserved syntenic blocks. (PDF 458 kb) [file 12870_2016_716_MOESM4_ESM.pdf]
