# Supplementary material for: Functional and evolutionary analyses of the miR156 and miR529 families in land plants
Source: BMC Plant Biol. 2016 Feb 3;16:40. doi: 10.1186/s12870-016-0716-5 (PMC4739381; doi:10.1186/s12870-016-0716-5)
Supplement: Additional file 5: — PARE and RNA-seq data for rice MIR156 and MIR529 precursors. (PDF 58 kb) [file 12870_2016_716_MOESM5_ESM.pdf]

Additional file 5. PARE and RNA-seq data for rice *MIR156* and *MIR529* precursors.

miRNA precursors	PARE libraries: Three-week-old wild-type seedlings (SC938)[a]	PARE libraries: Wild-type young panicle (INF939)	PARE libraries: Wild-type young inflorescences (4-6cm; INF9311a)	PARE libraries: Three-week-old wild-type seedlings (NPBs)	Total	RNA-seq library: Wild-type leaf tissue[b]	sRNA-seq library: Wild-type leaf tissue[b]
MIR156a	23	49	1	97	170	n.d.	<15,000
MIR156b	23	49	1	97	170	<10	<15,000
MIR156c	23	49	1	103	176	<10	<15,000
MIR156d	34	109	3	257	403	>10	>80,000
MIR156e	23	49	1	73	146	n.d	<15,000
MIR156f	31	126	24	197	378	>10	>80,000
MIR156g	23	49	7	113	192	>10	<15,000
MIR156h	37	120	4	199	360	>10	>80,000
MIR156i	23	49	1	78	151	n.d	<15,000
MIR156k	0	3	1	4	8	n.d	<100
MIR156l	0	0	3	14	17	n.d	<100
MIR529a	0	0	69	40	109	n.d	<15,000
MIR529b	0	0	0	10	10	n.d	<100

[a]Sequence abundance for all PARE samples was normalized to TP10M (transcript per 10 million reads). Data were retrieved from publicly available database (https://mpss.udel.edu/dbs/index.php?SITE=rice_pare).

[b]Sequence abundance from RNA-seq and sRNA-seq libraries was normalized to TP10M. Data were retrieved from Stroud et al. (2013). N.d, not detected.

Reference:

Stroud H, Ding B, Simon SA, Feng S, Bellizzi M, Pellegrini M, Wang GL, Meyers BC, Jacobsen SE. 2013. Plants regenerated from tissue culture contain stable epigenome changes in rice. Elife. 2:e00354.
