# Supplementary material for: Functional and evolutionary analyses of the miR156 and miR529 families in land plants
Source: BMC Plant Biol. 2016 Feb 3;16:40. doi: 10.1186/s12870-016-0716-5 (PMC4739381; doi:10.1186/s12870-016-0716-5)
Supplement: Additional file 6: — Oligonucleotide sequences used in this work. (PDF 84 kb) [file 12870_2016_716_MOESM6_ESM.pdf]

Additional file 6. Oligonucleotide sequences used in this work.

**Oligonucleotide**	**Gene**	**Sequence**	**Use**
AtSPL9-F	*AtSPL9*	GGTCGGGTCAGTCGGGTCAGATACC	RT-PCR
AtSPL9-R	*AtSPL9*	ACTGGCCGCCTCATCACTCTTGTATCC	RT-PCR
AtSPL15-F	*AtSPL15*	AGAAGCAAGAACCGGGTCAATACC	RT-PCR
AtSPL15-RNested	*AtSPL15*	AGCCATTGTAACCTTATCGGAGAATGAG	RT-PCR
AtSPL15- R	*AtSPL15*	TCATCGAGTCGAAACCAGAAGAT	RT-PCR
AtSPL13-F	*AtSPL13*	CCAATCTCTTCTTCTCCAAACAGTACCAGAAGC	RT-PCR
AtSPL13-R	*AtSPL13*	GAAGCAAATGAGGGACTGACGACG	RT-PCR
AtSPL10-F	*AtSPL10*	GTGGGAGAATGCTCAGGAGGC	RT-PCR
AtSPL10-R	*AtSPL10*	GAGTGTGTTTGATCCCTTGTGAATCC	RT-PCR
AtSPL6-F	*AtSPL6*	CTTGGAGCTACGGGAGAAGC	RT-PCR
AtSPL6-R	*AtSPL6*	TCCATTGGAGTTACCAGCCA	RT-PCR
AtSPL5-F	*AtSPL5*	ATGCAGCAGGTTTCATGAGC	RT-PCR
AtSPL5-F	*AtSPL5*	GCCTGACCCTTCTCCAAAAC	RT-PCR
AtSPL4-R	*AtSPL4*	CCAAAATGGAGGGTAAGAGA	RT-PCR
AtSPL4-R	*AtSPL4*	GCCTCTTTCATATCAGCTGTGC	RT-PCR
AtSPL3-F	*AtSPL3*	CTTAGCTGGACACAACGAGAGAAGG	RT-PCR
AtSPL3-R	*AtSPL3*	GAGAAACAGACAGAGACACAGAGGA	RT-PCR
AtSPL2-F	*AtSPL2*	CCGACGTCTCTCAGATCACA	RT-PCR
AtSPL2-R	*AtSPL2*	TGGTACGTGCTTCGAACTTG	RT-PCR
Os529pre-F	*OsMIR529* precursor	CTCCCTCTTCTTCTCTTAGC	RT-PCR

Additional file 6. **Continued**

**Oligonucleotide**	**Gene**	**Sequence**	**Use**
Os529pre-R	*OsMIR529* precursor	GGCTGCATGGACAGATAAGA	RT-PCR
AtActina –F	*AtACTIN-2*	GACCTTGCTGGACGTGACCTTAC	RT-PCR
AtActina –R	*AtACTIN-2*	GTAGTCAACAGCAACAAAGGAGAGC	RT-PCR
OsSPL14 -F	*OsSPL14*	ATCTCCGGTGGTATCCAGTG	RT-PCR
OsSPL14 -R	*OsSPL14*	CACGATGGATTGGTCTCTGTA	RT-PCR
OsUbiquitina –F	*OsUBIQUITIN*	AGAAGGAGTCCACCCTCCACC	RT-PCR
OsUbiquitina -R	*OsUBIQUITIN*	GCATCCAGCACAGTAAAACACG	RT-PCR
OsmiR529-F	OsmiR529b	CCTGAGAGAAGAGAGAGAGTA	RT-PCR
Stem-loop OsmiR529	OsmiR529b	GTCGTATCCAGTGCAGGGTCCGAGGTATTCGCACTGGATACGACAGGCTG	RT-PCR
AtmiR156-F	AtmiR156	CCTGAGTGACAGAAGAGAGTG	RT-PCR
Stem-loop AtmiR156	AtmiR156	GTCGTATCCAGTGCAGGGTCCGAGGTATTCGCACTGGATACGACTGCTCT	RT-PCR
Aqc529pre-F	*AqcMIR529* precursor	CACCGGATCCAGAGATGAGAGATATGTGACAGAAG	RT-PCR
Aqc529pre-R	*AqcMIR529* precursor	GCAGAGCTCAGAGAGAGAGAGATTTGGTGATGA	RT-PCR
Reverso universal		GTGCAGGGTCCGAGG	RT-PCR
OsMIR529b_F	p35S::OsMIR529b	CACCGGATCCTAGTACTAAGTTGAGTTCCATCCGA	Construction
OsMIR529b_R	p35S::OsMIR529b	GCAGAGCTCCTGTCATTAGTTAAAACTAGAACATGC	Construction
AqMIR529_F	p35S::AqcMIR529	CACCAGAGATGAGAGATATGTTGACAGAAG	Construction
AqMIR529_R	p35S::AqcMIR529	GCAAGAGAGAGAGAGATTTGGTGATGA	Construction
