# Supplementary material for: Functional and evolutionary analyses of the miR156 and miR529 families in land plants
Source: BMC Plant Biol. 2016 Feb 3;16:40. doi: 10.1186/s12870-016-0716-5 (PMC4739381; doi:10.1186/s12870-016-0716-5)
Supplement: Additional file 7: — Multiple sequence alignment of selected SPL nucleotide sequences. (PDF 86 kb) [file 12870_2016_716_MOESM7_ESM.pdf]

Additional file 7. **Multiple Sequence Alignment of Selected *SPL* nucleotide sequences.** Multiple sequence alignment was performed using the MUSCLE algorithm (Edgar, 2004).

```
OsSPL2          CGGCGTGCTCGGTGGAGGGGTGCGCCGCCGACCTGTCCAAGTGCGTCGACTACCACCGGC
PtSPL1          CCTTTTGCCAGGTCTATGATTGTAACAAGGATCTTAGTTCCTCCAAGGATTACCACAAGA
PpSPL3          CGCGTTGTCAAGTGCAGGGTTGTGATGCCGACCTGAGCTGTTGCAAAGACTATCACAAAC
PpSPL6          CGCGTTGTCAAGTCGAGGGCTGTAAGGCTGATCTAAGTGGTTGCAAAGACTATCACAAGC
PpSPL13         CGCGTTGTCAAGTGGAGGGCTGTAAGGCTGATCTGAGTGGTTGCAAAGATTATCATAAGC
ZmSBP7          CGCGCTGCCTGGTCGACGGCTGCAACGCCGACCTCACCGACGCCAAGACCTACTACTGCC
ZmSBP5          CCCGCTGCCAGGTCGACGGCTGCAACGTTGACCTCACCGACGTCAAGGCCTACTACTGCC
SbSPL5          CGCGCTGCCAGGTCGACGGCTGCAACGTGGACCTCACCGACGTCAAGCCCTACTACTGCC
BdSPL14         CGCGGTGCCAGGTGGAAGGGTGCGAAGTGGATCTCACGGCCTCCAAGGGCTACTACTGCC
OsSPL14         CGCGGTGCCAGGTGGAGGGGTGCGGCGCGGATCTGAGCGGGATCAAGAACTACTACTGCC
BdSPL17         CGAGGTGCCAGGTGGAAGGGTGCGGCGTGGATCTGAGCGGCGACAAGACCTACTACTGCC
ZmSBP1          CGCGGTGCCGGGTGGACGGGTGCGGCGTGGATCTGAGCGCCGTCAAGCAGTACTACTGCC
TSH4            CGAGGTGCCAGGTGGACGGGTGCGGCGTGGATCTGAGCGCCGTCAAGCAGTACTACTGCC
AlSPL           CGAGGTGCCAAGTGGAAGGTTGTAGAATGGATCTAAGCAATGTAAAAGCTTATTACTCAA
AtSPL15         CGAGGTGCCAAGTGGAAGGTTGTAGAATGGATCTAAGCAATGTTAAAGCTTATTACTCGA
AtSPL9          CAAGGTGCCAAGTGGAAGGTTGTGGGATGGATCTAACCAATGCAAAAGGTTATTACTCGA
ThSPL           CAAGGTGCCAAGTGGAAGGTTGTGGGATGGATCTAACCAATGCAAAGGGTTATTACTCTA
SlSBP15         CTAGGTGCCAAGTTGAAGGTTGTCAGGCAGATCTGAGTGATGCTAAGGCTTACTATTCTA
MtSPL           CTAGGTGTCAAGTTGAAGGATGTAAACTAGATCTGACTGATGCTAAAGCTTACTATTCTA
MdSPL1          CGCGGTGTCAGGTGGAGGGCTGCGAGGTAGATCTGAGTGGTGCCAAAGCTTACTATTCCA
AmSPL4          CTAGGTGCCAGGTGGAGGGTTGTAAGATAGATCTGAGTGATGCTAAGGCTTATTATTCAA
GmSPL9          CTAGGTGTCAGGTTGAGGGCTGCAAAGTAGATCTGAGTGGTGCTAAGGCTTACTATTCTA
PontSPL9        CAAGGTGCCAAGTGGAGGGGTGTAAAGTTGATCTGAGTGATGCCAAAGCTTACTATTCAA
VvSPL9          CGAGGTGTCAAGTTGAAGGGTGTAAAGTAGATCTGAGTGATGCCAAAGCTTACTATTCAA
PtSPL2          CAAGGTGTCAAGTTGAAGGGTGCAAAGTAGATCTGAGTGATGCTAAGACTTACTATTCAA
RcSPL           CTAGGTGTCAAGTTGAAGGGTGTAAAGTGGATCTGAGTGATGCTAAGGCTTACTATTCAA
                *    **    **  * *  **       ** **                **  *

OsSPL2          GGCACAAGGTGTGCGAGGCGCACTCCAAGACGGCCGTCGTCACCGTCGCCGGCCAGCAGC
PtSPL1          GGCATAAAGTTTGTGAGGTTCATACGAAAACTCCTCAAGTTATTGTTAATGGCAATGAAC
PpSPL3          GTCACAAAGTGTGTGAGATGCACTCCAAAGCGGCCACAGCAATAGCAGCTGGAATTGAGC
PpSPL6          GTCACAAGGTTTGCGAGATGCACTCCAAAGCGCCCAAATGCATTGCAGCTGGGATTGAGC
PpSPL13         GTCACAAGGTGTGTGAGATGCACTCCAAGGCGCCCAAATGCATTGCAGCTGGGATTGAGC
```

```
ZmSBP7          GCCACAAGGTGTGCGAAATGCACTCCAAGGAGCCCCGCGTCGTCGTCAACGGTCTCGAGC
ZmSBP5          GCCACAAGGTGTGCAAAATGCACTCCAAGGAGCCCCGCGTCCTCGTCAACGGCCTCGAGC
SbSPL5          GCCACAAGGTCTGCAAAATGCACTCCAAGGAGCCCCGCGTCGTCGTCAACGGCCTCGAGC
BdSPL14         GCCACAAGGTGTGCTCCATGCACGCCAAGTCGCCCCGCGTCGTCGTCGCCGGCCTCGAGC
OsSPL14         GCCACAAGGTGTGCTTCATGCATTCCAAGGCTCCCCGCGTCGTCGTCGCCGGCCTCGAGC
BdSPL17         GCCACAAGGTGTGCTCCATGCACTCCAAGGCGCCGCTCGTCGTCGTCGCCGGCATCCAGC
ZmSBP1          GGCACAAGGTGTGCTACATGCACTCCAAGGAGCCGCGCGTCTTCGTCGCCGGCATCGAGC
TSH4            GGCACAAGGTGTGCAACATGCACTCCAAGGAGCCGCGCGTCTTCGTCGCCGGCATCGAGC
AlSPL           GACACAAAGTTTGTTGCATTCACTCTAAATCATCTAAAGTCATTGTCTCTGGTCTTCATC
AtSPL15         GACACAAAGTTTGTTGCATTCACTCTAAATCATCTAAAGTCATTGTCTCTGGTCTTCATC
AtSPL9          GACACCGAGTTTGTGGAGTGCACTCTAAAACACCTAAAGTCACTGTGGCTGGTATCGAAC
ThSPL           GGCACAGAGTTTGTGGAATACACTCTAAAACACCCAAAGTCATTGTCGCTGGTCTCGAAC
SlSBP15         GGCATAAAGTTTGTGGTATGCACTCTAAGTCTCCTACTGTTGTTGTTGCTGGTCTTGAAC
MtSPL           GACACAAAGTTTGTAGCATGCACTCTAAATCCCCTACTGTTACTGTTTCTGGTCTTCAAC
MdSPL1          GGCACAAAGTCTGTGGCTTGCACTCTAAAACTCCCACTGTCATTGTTGCTGGTCTTGAAC
AmSPL4          GGCACAAAGTTTGTGGTATGCATTCAAAGTCTCCAAAGGTCATTGTTGCAGGAATTGAAC
GmSPL9          GGCACAAAGTTTGTACCATGCACTCCAAGTTCCCTACTGTTATTGTTGCTGGTTTGGAGC
PontSPL9        GGCACAAAGTTTGTGGCATGCATTCAAAGTCTCCTGTTGTCACTGTTGCCGGCCTTGAGC
VvSPL9          GGCATAAAGTGTGTGGTATGCATTCGAAGTCTCCAACGGTCATTGTTGCGGGCCTTGAGC
PtSPL2          GGCACAAAGTTTGTAGTATGCACTCCAAGTCTCCTAGAGTTATTGTTGCTGGTTTGGTGC
RcSPL           GGCATAAAGTTTGTGGTATGCACTCTAAGTCTCCTAAGGTCATTGTTGCTGGTTTGGAAC
                * **    ** **       **  * **     *          *     **       *

OsSPL2          AGCGCTTCTGCCAACAGTGCAGCAGGTTTCATTTGCTCGGGGAGTTCGATGAGGAGAAGA
PtSPL1          AGAGGTTTTGTCAGCAGTGTAGCAGGTTTCACTTGCTGGTTGAGTTTGATGATGGTAAGC
PpSPL3          AGCGTTTCTGCCAACAATGTTCGAGATTTCATGTGCTGAAGGAATTTGATGAAGGAAAAC
PpSPL6          AGCGTTTCTGCCAACAGTGTTCGAGGTTTCATGTCCTCACCGAGTTCGACGAAGGGAAGC
PpSPL13         AGCGTTTCTGTCAACAGTGTTCGAGGTTCCATGTCCTGACTGAGTTCGATGAAGGGAAAC
ZmSBP7          TACGCTTCTGCCAGCAGTGCAGCAGGTTCCACAACCTGGCCGAATTTGACCAGCAAAAGA
ZmSBP5          AGCGCTTCTGCCAGCAGTGCAGCAGGTTCCACCAGCTGCCTGAATTCGACCAGCTAAAGA
SbSPL5          AGCGCTTCTGCCAGCAGTGCAGCAGGTTCCACCAGCTGCCTGAATTTGACCAACTAAAAA
BdSPL14         AGCGCTTCTGCCAGCAGTGCAGCAGGTTCCACCAGTTGCCTGAATTTGATCAAGGAAAAC
OsSPL14         AGCGCTTCTGCCAGCAGTGCAGCAGGTTCCACCTGCTGCCTGAATTTGACCAAGGAAAAC
BdSPL17         AGCGCTTCTGCCAACAGTGCAGCAGGTTCCACCAGTTACCTGAATTCGACCAAGGAAAAC
ZmSBP1          AGCGCTTCTGCCAGCAGTGCAGCAGGTTCCACCAGTTACCTGAATTTGACCAAGGGAAAC
TSH4            AGCGCTTCTGCCAACAGTGCAGCAGGTTCCACCAGCTACATGAATTTGACCAAGGGAAAC
AlSPL           AAAGGTTTTGCCAACAATGTAGCAGGTTTCACCAGCTTTCTGAGTTTGACTTAGAGAAAA
```

```
AtSPL15         AAAGGTTTTGTCAACAATGTAGCAGGTTTCACCAGCTTTCTGAGTTTGACTTGGAGAAAA
AtSPL9          AGAGGTTTTGTCAACAGTGCAGCAGGTTTCATCAGCTTCCGGAATTTGACCTAGAGAAAA
ThSPL           AGAGGTTTTGTCAACAGTGCAGCAGGTTTCATCAGCTTCCGGAGTTTGACCTAGAGAAAA
SlSBP15         AGAGGTTTTGCCAACAGTGTAGCAGGTTCCACCAATTAACTGAATTCGACCAGGGGAAAA
MtSPL           AAAGGTTTTGTCAACAATGTAGCAGATTTCATCAGCTTGCTGAGTTTGATCAAGGAAAAA
MdSPL1          AGAGGTTTTGCCAACAGTGTAGCAGGTTTCATTTACTTCCTGAATTTGATCAAGGAAAAC
AmSPL4          AAAGGTTTTGCCAGCAGTGCAGCAGGTTTCATCAATTGCCTGAATTTGACCAAGGAAAAC
GmSPL9          AAAGGTTTTGCCAACAGTGTAGCAGGTTTCATCTGCTTTCTGAATTTGATGAAGGAAAAC
PontSPL9        AGAGGTTTTGCCAGCAATGTAGCAGATTTCATCAGCTTCCGGAGTTTGACCAAGGAAAAC
VvSPL9          AGAGGTTTTGCCAGCAGTGTAGCAGATTTCATCAGCTTGCCGAATTTGACCAAGGAAAAC
PtSPL2          AAAGATTTTGCCAGCAATGTAGCAGATTTCATCTACTTCCTGAATTTGACCAAGGAAAAC
RcSPL           AAAGATTTTGCCAGCAGTGTAGTAGATTTCATCAACTTCCTGAATTTGACCAAGGAAAAC
                   * ** ** ** ** **    ** ** **     *    ** ** **       **

OsSPL2          GGAGCTGCAGGAAGCGGCTCGACGGGCACAACAAGCGGCGTCGGAAGCCGCACCTCGTAC
PtSPL1          GCAGTTGTCGTAAACGCCTGGCAGGCCACAATGAACGCAGACGAAAGCCTCGGGACTTCC
PpSPL3          GCAGTTGTCGTAGAAGACTAGCAGGTCATAATCAACGGCGACGGAAGTCTCGCATCCTCA
PpSPL6          GCAGCTGTCGCAGGAGATTGGCAGGTCACAATGAACGGCGAAGAAAGCCACGCATCGTCA
PpSPL13         GCAGTTGTCGTAGAAGGCTGGCAGGTCACAATGAGCGGCGCAGGAAGCCTCGCATCGTCA
ZmSBP7          AAAGCTGTCGCAAGCGTCTAGCAGGCCACAACGAACGCCGGAGACGGCCACGAAGCTTCA
ZmSBP5          AGAGCTGCCGCAAACGCCTCGCAGGCCACAACGAGCGCCGGAGGAGGCCGCGAAGCTTCA
SbSPL5          AAAGCTGCCGCAGACGTCTCGCAGGCCACAATGAACGCCGGAGGAGGCCACGAAGCTTTA
BdSPL14         GCAGCTGCCGCAGACGCCTCGCAGGCCACAATGAGCGCCGCAGGAGGCCACGGAGCTATC
OsSPL14         GCAGCTGCCGCAGACGCCTTGCAGGTCATAATGAGCGCCGGAGGAGGCCGCGAAGCTTTA
BdSPL17         GAAGTTGCCGCAGGCGCCTAGCGGGTCACAATGAGCGCCGGAGGAAGCCCCGAAGCTTTC
ZmSBP1          GAAGCTGCCGCCGCCGCCTCATTGGTCACAACGAGCGCCGGAGGAAGCCACGGAGCTTCC
TSH4            GTAGCTGCCGCCGCCGCCTCATCGGTCACAACGAGCGCCGGAGGAAGCCACGAAGCTTCC
AlSPL           GAAGTTGTCGCAGACGACTCGCTTGTCATAACGAACGTCGAAGAAAGCCACAAAGCGTTT
AtSPL15         GAAGTTGTCGCAGAAGACTCGCTTGTCATAACGAACGACGAAGAAAACCACAAAGCGTTT
AtSPL9          GGAGTTGCCGCAGGAGACTCGCTGGTCATAATGAGCGACGAAGGAAGCCACGAAGCTTTC
ThSPL           GAAGTTGCCGTAGGAGACTCGCTGGCCATAATGAGCGTCGGAGAAAGCCACGGAGCTTTC
SlSBP15         GGAGTTGCCGCAGGAGACTGGCATGCCATAATGAGCGTCGTAGGAAGCCTCGAAGCTTTC
MtSPL           GAAGTTGTCGGAGACGACTAGCTGGTCATAATGAGCGTCGCAGAAAGCCCCGCAGCTTTT
MdSPL1          GTAGTTGTCGTAGACGCTTGGCTGGGCATAATGAGCGTCGTAGAAAACCACGAAGCTTTC
AmSPL4          GAAGTTGCCGCAGACGCCTTGCTGGCCACAATGAGCGTCGGAGGAAGCCGCGAGGCTTCG
GmSPL9          GAAGCTGCCGCAGGCGACTTGCTGGTCATAATGAGCGCCGACGAAAGCCCCGTGGCTTTC
PontSPL9        GAAGTTGCCGCAGGCGCCTGGCAGGCCATAATGAGCGCCGGAGGAAGCCAAGAGGATTTC
```

```
VvSPL9          GAAGTTGTCGTAGGCGCCTGGCTGGTCATAATGAGCGTCGCAGGAAGCCACGAGGCTTTC
PtSPL2          GAAGTTGCCGCAGGCGCCTAGCTGGCCATAATGAGCGACGGAGGAAGCCACGAGGCCTTC
RcSPL           GAAGTTGTCGCAGACGCCTAGCAGGTCATAATGAGCGACGGAGGAAGCCACGAGGCTTTC
                  ** **  *     *  *     * ** **  * **  *  *     *
```

```
OsSPL2          CAACTGTGCTCTCTCTCTTCTGTCACTTTCTCATCGT
PtSPL1          TAGCTGTGCTCTCTCTCTTCTGTCAACTACTCTCTTT
PpSPL3          CGGCCGTGCTCTCTCTCTTCTGTCAAACAATCAATCT
PpSPL6          TGGTCGTGCTCTCTCTCTTCTGTCAAACAATCGATCT
PpSPL13         TGGCCGTGCTCTCTCTCTTCTGTCAAACAATCGACCT
ZmSBP7          TAGCTGTGCTCTCTCTCTTCTGTCAAGTGGTCGCTCT
ZmSBP5          TAGCTGTGCTCTCTCTCTTCTGTCAACTGGTCGCTCT
SbSPL5          TAGCTGTGCTCTCTCTCTTCTGTCAACTGGTCACTCT
BdSPL14         TAGCTGTGCTCTCTCTCTTCTGTCAACTGGTCTCAGT
OsSPL14         CAGCTGTGCTCTCTCTCTTCTGTCAATTGGTCTCTGT
BdSPL17         TAGCTGTGCTCTCTCTCTTCTGTCAACTGGTCTCTGT
ZmSBP1          TAGCTGTGCTCTCTCTCTTCTGTCAATTGGTCCCTGT
TSH4            TAGCTGTGCTCTCTCTCTTCTGTCAACTGGTCCCTGT
AlSPL           TAGCTGTGCTCTCTCTCTTCTGTCAATTGGTCTCTTT
AtSPL15         TAGCTGTGCTCTCTCTCTTCTGTCAATTGGTCTCTTT
AtSPL9          TAACTGTGCTCTCTCTCTTCTGTCAACTGGTCTCTCT
ThSPL           TAACTGTGCTCTCTCTCTTCTGTCAACTGGTCTCTCT
SlSBP15         TAGTGGTGCTCTCTCTCTTCTGTCAACTGGACTCTTT
MtSPL           TAACTGTGCTCTCTCTCTTCTGTCAAGTGGTCACTGT
MdSPL1          TAGCTGTGCTCTCTCTCTTCTGTCAACTGGTCACTTT
AmSPL4          TAGCGGTGCTCTCTCTCTTCTGTCAACTGGTCCCTAT
GmSPL9          TAACTGTGCTCTCTCTCTTCTGTCAACTGGTCTCTTT
PontSPL9        TAACTGTGCTCTCTCTCTTCTGTCAATTGGTCCCTTT
VvSPL9          TAGCTGTGCTCTCTCTCTTCTGTCAACTGGTCACTTT
PtSPL2          TAGCTGTGCTCTCTCTCTTCTGTCAACTGGTCACTTT
RcSPL           TTCCTGTGCTCTCTCTCTTCTGTCAACTGGTCGCTTT
                     ********************      *
```
